# High-Performance Impedance Humidity Sensor Based on Au Nanoparticle-Modified Hydroxyl-Rich Graphene Oxide

**DOI:** 10.1021/acsomega.5c03897

**Published:** 2025-09-19

**Authors:** Iuri K. Machado, Rafael de Oliveira, Marina C. Totti, Nayton C. Vicentini, Wesley W. G. Nascimento, Benjamin Fragneaud, Indhira O. Maciel, Cristiano Legnani, Antonio Carlos Sant’Ana, Welber G. Quirino

**Affiliations:** † Nanoscience and Nanotechnology Group, Department of Physics, Institute of Exact Sciences, Federal University of Juiz de Fora, Juiz de Fora, Minas Gerais 36036-900, Brazil; ‡ Plasmonic Nanostructures Laboratory, Department of Chemistry, Institute of Exact Sciences, Federal University of Juiz de Fora, Juiz de Fora, Minas Gerais 36036-900, Brazil; § Department of Pharmacy, Institute of Life Sciences, Federal University of Juiz de Fora, Governador Valadares Campus, Governador Valadares, Minas Gerais 35010-180, Brazil

## Abstract

This study explores the impedance response of hydroxyl-rich graphene oxide (HGO) modified with gold nanoparticles (AuNPs) for humidity sensing applications. The results demonstrate that the incorporation of AuNPs significantly enhances the electrical properties of HGO, improving the sensor’s overall performance. The sensor layer structure was systematically examined based on the order of material deposition—a topic rarely addressed in the literature. Our findings indicate that the initial deposition of AuNP directly onto the aluminum interdigitated film leads to superior sensing efficiency. The AuNP-modified HGO sensor exhibited a high sensitivity of 0.032 log Z/%RH and response times of approximately 0.2 s, highlighting the paramount synergy between both materials. Both HGO and AuNP/HGO sensors exhibited stable hysteresis behavior, with excellent adsorption response times and satisfactory desorption times, confirming their viability for humidity monitoring. Impedance analysis revealed distinct resistive and capacitive properties, with consistent capacitance across different humidity levels. We propose that proton conduction and ionic diffusion are the primary operating mechanisms, with the latter becoming more pronounced in the presence of AuNP, as evidenced by Warburg impedance behavior in Nyquist plots. Importantly, this study suggests that the sensor’s sensitivity is not solely due to water absorption but also enhanced by a reduced Schottky barrier resulting from interactions with adsorbed water molecules, thereby providing a broader perspective on the mechanisms influencing sensor performance. These insights were validated through various sensor preparation methods, including layer inversion during deposition.

## Graphical abstract

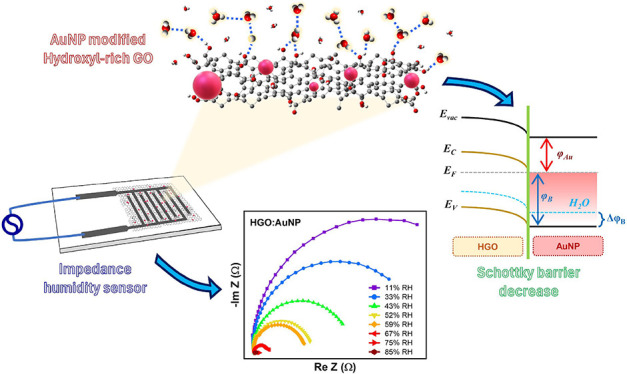

## 1 Introduction

Relative humidity (RH) sensors are critical in several sectors, including research laboratories, food, pharmaceutical, chemical, and textile industries, in addition to applications in medicine and agriculture, among others.^1–6^ In this context, the ongoing advancement of RH sensors to enhance their sensing properties is a promising and essential field of research. These advancements are driven by the use of materials with specific characteristics that improve device performance. Among these materials, carbon-based structures are particularly promising for the development of humidity sensors. However, challenges such as low sensitivity and conductivity, high hysteresis, suboptimal response, and recovery times can limit their effectiveness. For instance, while graphene-based sensors demonstrate high conductivity, they often exhibit limited sensitivity. Conversely, graphene oxide (GO), though less conductive, displays notable sensitivity due to its hydrophilic properties.^7–9^

Graphene oxide comprises a hexagonal crystalline structure with oxygenated functional groups (OFGs) on its surface due to chemical oxidation, including carbonyl, hydroxyl, epoxy, and carboxyl groups.^10^ The presence of OFGs disrupts the extended sp^2^ structure, altering its properties, increasing roughness, and enlarging its surface area, which makes GO-based materials ideal candidates for water sensing. Thus, controlling OFG content is essential for the performance of GO-based sensors in RH detection.^11–15^ Studies have shown that hydroxyl and epoxy groups contribute to enhanced conductivity, while carboxyl groups inhibit charge transfer.^16–18^ In this work, hydroxyl-rich graphene oxide (HGO) was synthesized via the modified Hummers’ method proposed by Chen et al.^19^ Hydroxyl groups were selected due to their strong interactions with water, primarily through hydrogen bonding, which enhances water adsorption and detection efficiency. Fatima et al.^13^ reported that hydroxyl group concentration significantly improves sensor performance.

Although extensive literature supports the importance of graphene oxide (GO) in relative humidity (RH) sensors,^20–24^ its high electrical resistance can often hinder electrical measurements. To address this limitation, research has focused on GO in combination with metallic nanomaterials such as copper (CuNP),^25,26^ silver (AgNP),^27,28^ and gold (AuNP)^29–32^ nanoparticles to enhance specific properties and overall performance. Su et al.^29^ examined the impedance behavior of a composite material comprising AuNP, GO, and 3-mercaptopropyltrimethoxysilane. Similarly, Li et al.^28^ developed a capacitive sensor incorporating GO and AgNP, exploring the relationship between impedance and material interaction. Both studies highlight the importance of incorporating nanoparticles to enhance electrical conductivity and sensitivity, thereby improving sensor efficiency. Specifically, these works showed that the inclusion of metal nanoparticles significantly reduced the impedance of the composite materials and increased their responsiveness to humidity variations, resulting in faster response and recovery times as well as enhanced signal stability. These findings further support the strategy adopted in this work in which AuNPs are combined with hydroxyl-rich graphene oxide.

Nanostructured metals have been widely adopted due to their extensive applicability across various fields, owing to their unique optical and chemical/structural characteristics, including biocompatibility, a high surface-area-to-volume ratio, chemical stability, and high thermal and electrical conductivity. These properties make them particularly valuable in catalytic applications, both independently and in combination with other materials.^33–35^ Few studies have investigated this combined effect in humidity sensors. This study compares the performance of sensors containing only HGO with those integrated with nanoparticles, allowing for a thorough examination of the interaction between these materials and their effect on sensor efficiency. Moreover, this is the first work to evaluate the deposition of these two nanomaterials in various configurations, revealing notable differences that directly influence sensor performance.

The device fabrication process began with the deposition of aluminum onto the substrate, resulting in the formation of the interdigitated electrode, as shown in Figure 1A. Subsequently, four distinct sensor architectures were developed, as illustrated in Figure 1B. The first sensor consists solely of HGO; the second is a bilayer structure with HGO at the bottom and AuNP on top (HGO/AuNP); the third involves a homogeneous mixture of both components (HGO:AuNP); and the fourth is a bilayer with AuNP deposited first, followed by HGO (AuNP/HGO). These different fabrication strategies enabled a systematic evaluation of how the arrangement of materials affects the electrical properties and sensing performance.

**Figure 1:**
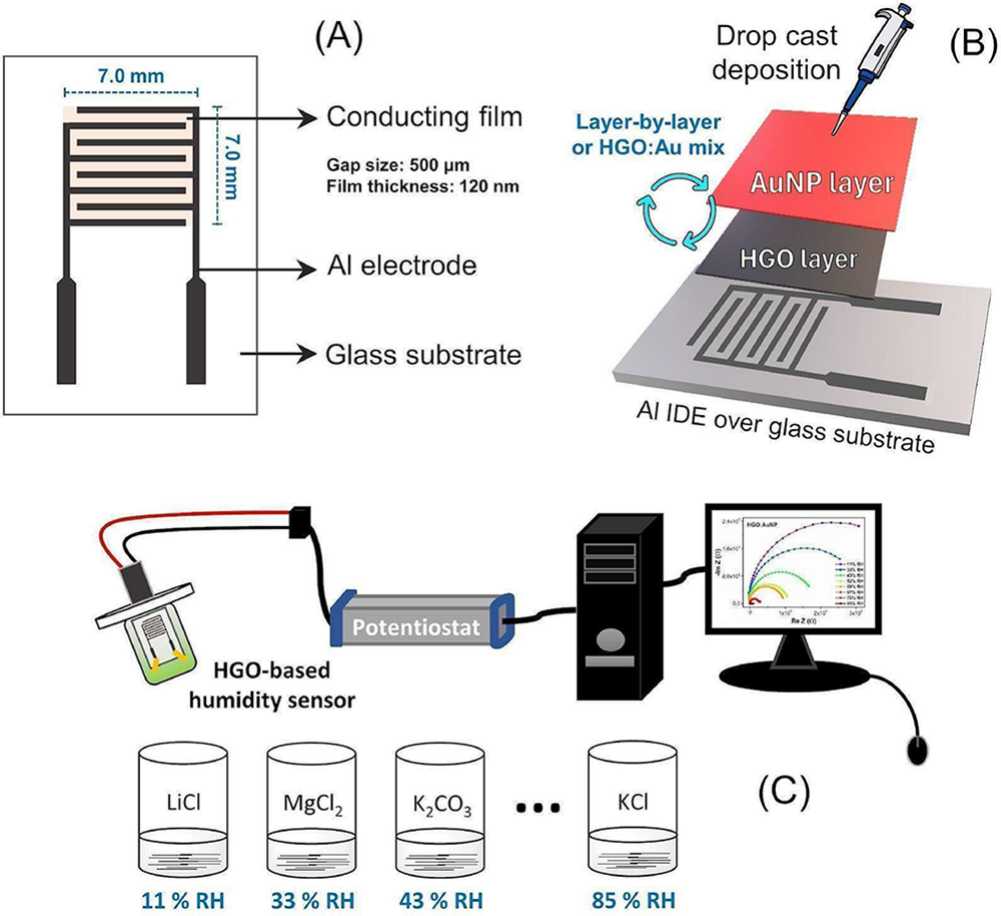
(A) Structure of the Al interdigitated electrode. (B) Schematic representation for humidity sensor fabrication. (C) Schematic representation of the apparatus used in the electrical characterization of all humidity sensors.

To characterize the sensors, each device was exposed to different humidity environments using eight hermetically sealed containers, each containing a saturated saline solution that generates a specific relative humidity (RH) at room temperature (25 °C): LiCl (11%), MgCl_2_ (33%), K_2_CO_3_ (43%), Mg­(NO_3_)_2_ (52%), NaBr (59%), CuCl_2_ (67%), NaCl (75%), and KCl (85%).^36–41^ Each sensor was mounted on a printed circuit board connected to a potentiostat and placed inside the container between the lid and the solution interface. The electrical connections passed through the lid and were sealed with epoxy resin, as shown in Figure 1C. After RH stabilization, an alternating voltage of 0.5 V with a 10 mV amplitude was applied over a frequency range of 10^2^ to 10^6^ Hz. Additionally, dynamic response and recovery times to humidity variations were evaluated under ambient conditions using human breath as a transient source of moisture.

Additionally, real-time impedance measurements at different humidity levels and repeatability tests were conducted to complement the characterization of sensor performance. These experiments were performed by using an excitation voltage of 0.5 V, an amplitude of 150 mV, and a fixed frequency of 100 Hz.

In this work, we fabricated four types of sensors: one containing only HGO and three others with added AuNPs. Various techniques were used to characterize the structural and optical properties of these humidity sensors including Raman scattering spectroscopy, Fourier-transform infrared absorption spectroscopy (FT-IR), ultraviolet–visible spectroscopy (UV–vis), scanning electron microscopy (SEM), and atomic force microscopy (AFM). Subsequently the sensors were evaluated using electrical impedance spectroscopy, with relative humidity ranging from 11 to 85%. Operating at an optimized voltage, these devices exhibited excellent water detection performance. The key performance parameters investigated in this work include sensitivity, which quantifies the sensor’s ability to respond to variations in relative humidity; hysteresis, defined as the difference in response between increasing and decreasing humidity cycles; linearity, which refers to the linear correlation between the logarithm of impedance and relative humidity; and response and recovery times, which describe the time the sensor takes to reach a steady-state impedance upon exposure to or removal of humidity, respectively.

## 2 Results and Discussion

Figure 2A presents the UV–vis spectra for pure AuNP, HGO, and their mixture. The HGO spectrum displays a maximum absorption band at 236 nm, associated with π → π* electronic transitions representing C=C bonds within the aromatic rings. Additionally, a shoulder band at 300 nm is observed, attributed to *n* → π* transitions related to oxygen-functional groups (OFGs) such as carbonyl and carboxyl moieties present in the GO structure.^42^ The localized surface plasmon resonance (LSPR) band at 515 nm is observed for the AuNP sample, indicating small particle size and narrow size distribution. This is consistent with previous findings by Assari et al.^30^ and Suchomel et al.,^43^ and it is further corroborated by the DLS analysis presented in Figure 2B, which confirms a narrow hydrodynamic size distribution centered at approximately 18 nm.

**Figure 2:**
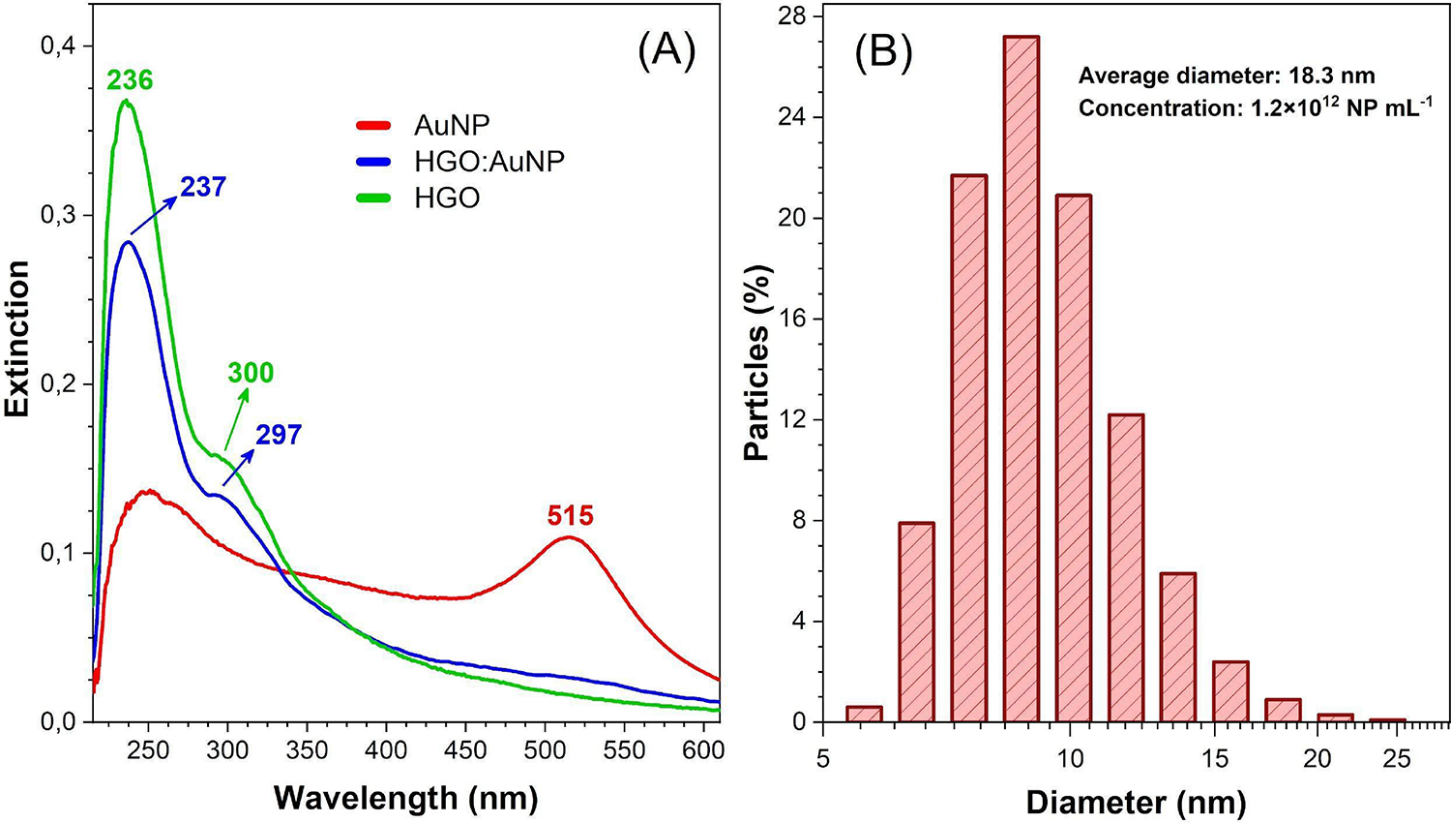
(A) UV–vis spectra of the HGO, AuNP, and HGO:AuNP samples. The hybrid HGO:AuNP film shows combined spectral features of the individual components. (B) Dynamic light scattering (DLS) analysis of AuNP, showing a mean hydrodynamic diameter of 18.3 ± 1.7 nm, indicating a narrow size distribution.

Figure 3A shows the Raman spectra of the humidity sensors. In the HGO sensor spectrum, two characteristic bands are present: the D-band at 1346 cm^–1^, primarily attributed to structural defects, and the G-band at 1600 cm^–1^, associated with C–C stretching modes within the sp^2^ graphitic structure. The intensity ratio of the D- and G-bands (*I*_D_/*I*_G_), which can be used to assess the quality or OFG concentration on the GO surface, was estimated to be 1.17 ± 0.02. This ratio, when compared to the width of the G-band, indicates a high level of defects^44^ and a high concentration of OFGs. This result is consistent with the expected properties of hydroxyl-rich carbon-based materials and supports their suitability for humidity sensing applications, due to their enhanced hydrophilicity and surface activity, which promote water molecule adsorption.^45^ In the spectra of sensors prepared via layer-by-layer deposition of HGO and AuNP, the D- and G- bands were also observed, even though slightly shifted, with minor variations in the *I*_D_/*I*_G_ ratios. These ratios (1.22 ± 0.03 for HGO/AuNP, 1.21 ± 0.02 for AuNP/HGO, and 1.22 ± 0.03 for the HGO:AuNP mix) do not indicate significant differences between sensors.

**Figure 3:**
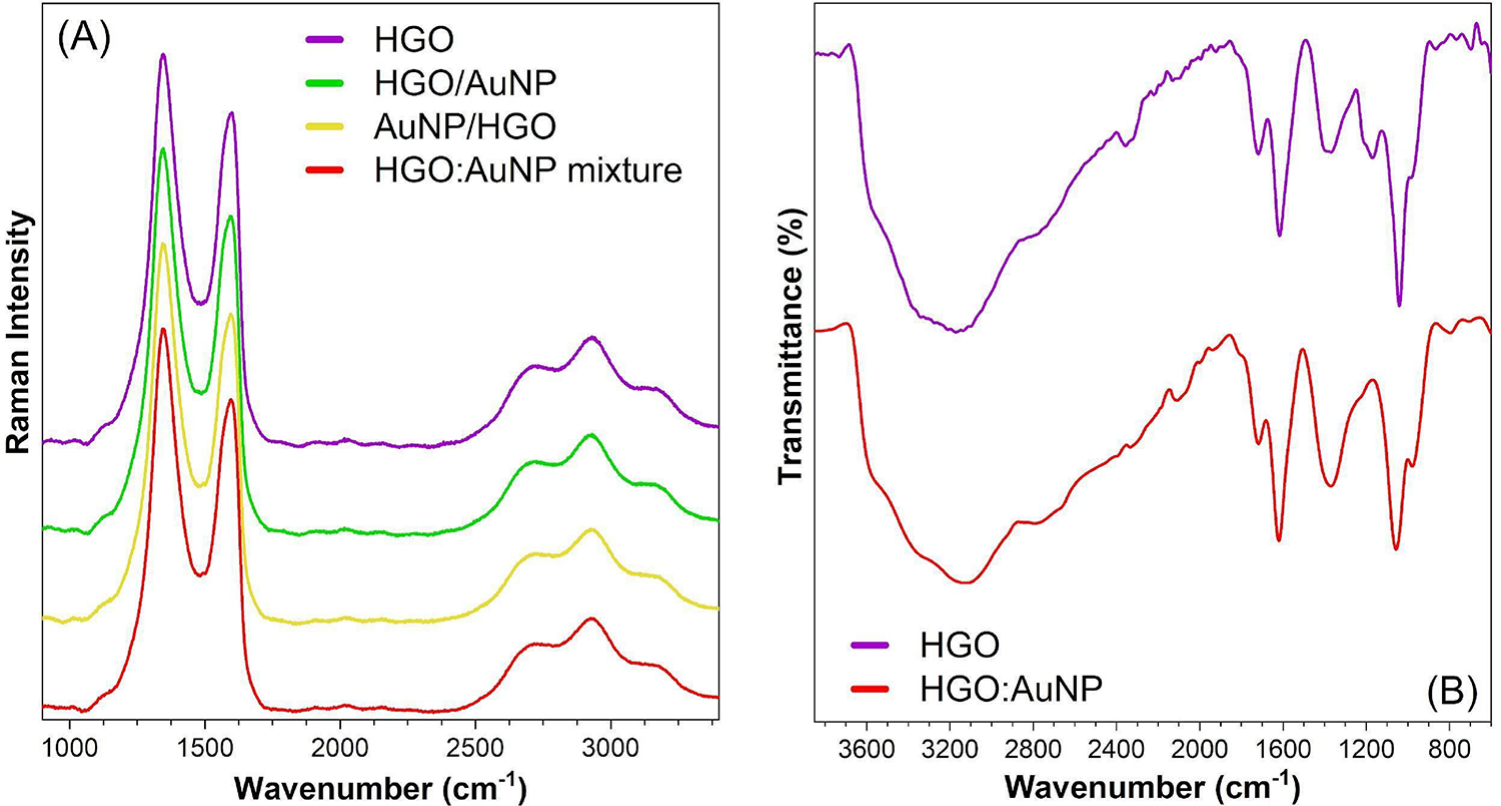
(A) Raman spectra of humidity devices produced with HGO and the different HGO/AuNP mixtures. (B) FT-IR spectra of HGO and the HGO:AuNP mixture.

Figure 3B presents FT-IR spectra of pure HGO and the HGO/AuNP mixture. The broad and intense band between 3700 and 3000 cm^–1^ is attributed to O–H stretching modes primarily from hydroxyl groups, with contributions from carboxylic groups, overlapping with C–H stretching modes between 3100 and 2600 cm^–1^. The band a narrow at 1718 cm^–1^ is assigned to C=O stretching modes of the carbonyl mode. The band at 1620 cm^–1^, according to several studies in the literature, is attributed to stretching vibrations of C=C bonds in GO samples. However, a recent study by Brusko et al. showed that this band is associated with bending vibrations of adsorbed water. In addition, the C=C stretching mode of unoxidized carbon appears in the range of 1585–1570 cm^–1^ and is superimposed on the band originated by bending vibrations of water. Figure 3B presents FT-IR spectra of pure HGO and the HGO/AuNP mixture. The broad and intense band between 3700 and 3000 cm^–1^ is attributed to O–H stretching modes, primarily from hydroxyl groups, with contributions from carboxylic groups, overlapping with C–H stretching modes between 3100 and 2600 cm^–1^.^10^ The narrow band at 1718 cm^–1^ is assigned to C=O stretching modes of the carbonyl mode.^46^ The band at 1620 cm^–1^, according to several studies in the literature, is attributed to stretching vibrations of the C=C bonds in GO samples. However, a recent study by Brusko et al. showed that this band is associated with bending vibrations of adsorbed water.^47^ In addition, the C=C stretching mode of unoxidized carbon appears in the range of molecules.

The C–OH bonds of the hydroxyl groups present deformation and stretching frequencies, evidenced by the absorption bands at 1377 cm^–1^.^48,49^ The bending vibrations of the C–O–H fragment of the carboxyl groups are detected as a shoulder band at 1222 cm^–1^, while the bands at 1056 and 980 cm^–1^ correspond to the C–O vibrations in the hydroxyl and epoxy groups.^49,50^ Some subtle band shifts are observed throughout the spectrum, mainly in bands ascribed to vibrations involving oxygen atoms, which are possible sites of interaction between the metal nanoparticles and HGO. This observation is further corroborated by the increase in transmittance of the band at 1377 cm^–1^ in the pure HGO spectrum and its shift to 1373 cm^–1^ in the modified material. This shift is attributed to the interaction of AuNPs with hydroxyl groups—the dominant oxygenated functionalities in the structure.^51^ Therefore, the increase in transmittance is expected and is interpreted as a positive indication of successful chemical interaction and functionalization, which can contribute to improved electrical properties and enhanced humidity sensing performance.

Overall, the spectrum of the HGO/AuNP mixture shows a profile similar to that of HGO, with slight band broadening and shifts, along with an increase in transmittance, as previously indicated by Raman spectroscopy. Additionally, the presence of OFGs on the GO surface is critical for detecting water molecules as these groups can form hydrogen bonds and enhance attraction to analyte molecules.

Figure 4A,B shows SEM images at different magnifications of the AuNP/HGO sensor surface. Although the aluminum substrate is not directly visible, the imaging reveals the surface morphology of the HGO film deposited over it. The bright spots observed are identified as clusters of Au nanoparticles due to their spherical morphology, relatively uniform size, and high electron density—features that generate increased brightness and contrast under SEM imaging. Their distribution is consistent with the expected pattern resulting from the drop-casting process, further supporting this identification. Figure 4C shows the surface roughness of the AuNP/HGO film obtained via AFM, with an RMS value of 5.52 nm. This relatively low roughness suggests a good level of homogeneity in the film morphology, which is beneficial for ensuring uniform electrical behavior. The RMS roughness value was calculated from the AFM topography by determining the standard deviation of the surface height variations within the scanned area.

**Figure 4:**
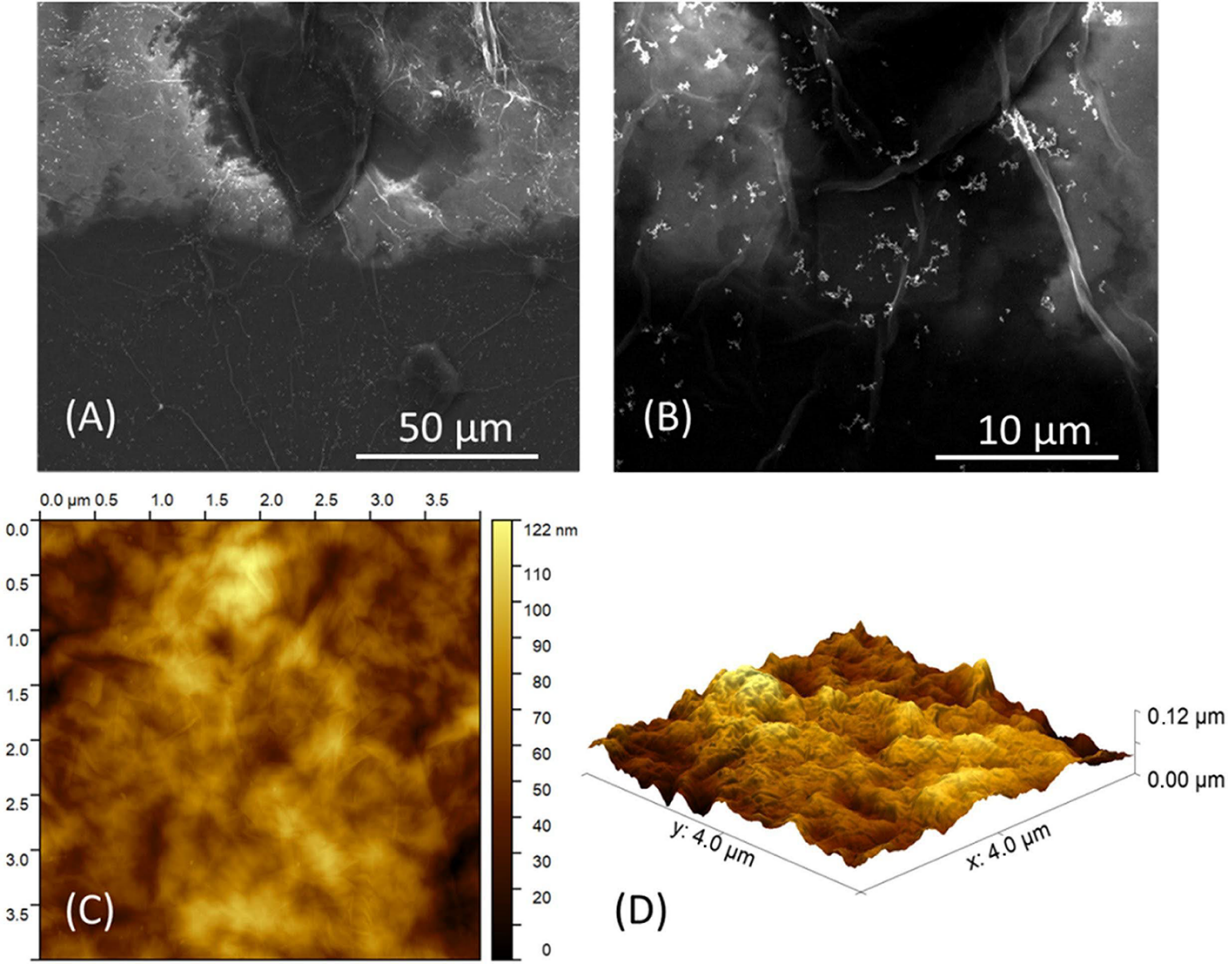
SEM images of the HGO/AuNP sensor at (A) 2 k× and (B) at 10 k× magnification. (C) 2D and (D) 3D AFM images of an area of 16.0 μm^2^ in the HGO/AuNP sensor.

Although RMS roughness and DLS hydrodynamic diameter describe different physical states of the material—AFM capturing the morphology of the dried film and DLS assessing the nanoparticle size in suspension—they are indirectly related. A stable and well-dispersed suspension, as indicated by the narrow size distribution in DLS (Figure 2B), typically leads to smoother films after deposition, whereas aggregation in a suspension often results in higher surface roughness. Thus, the consistency between the DLS and AFM results supports the effective dispersion and integration of AuNPs within the HGO matrix.

Figure 5 shows Nyquist plots, where the imaginary impedance is plotted as a function of the real impedance of the sensors at eight different RH levels. The semicircular shape of the Nyquist plots indicates that the impedance response is primarily governed by charge transfer resistance (*R*_ct_) and double-layer capacitance at the interface between the sensing film and the humid environment, a typical feature of electrochemical systems. The decrease in the diameter of the semicircles with increasing relative humidity suggests a reduction in *R*_ct_, consistent with enhanced ionic conductivity due to water molecule adsorption on the sensing surface. The resistance at the beginning of the semicircle represents the contact resistance at the Al-GO interface, which remains constant with increasing humidity. The constancy of this component is crucial, as it indicates that contact resistance does not influence the electrical analysis, and the observed effects are primarily derived from the HGO/AuNP layer. The radius of the semicircle, attributed to sensor layer resistance, decreases as the RH increases for all four sensors. Notably, sensors incorporating AuNPs exhibit a more significant decrease in the semicircle radius with increasing RH when compared to the HGO sensor (Figure 5A), indicating enhanced charge transport due to the presence of metallic nanoparticles. Among these, the HGO/AuNP and HGO:AuNP sensors (Figure 5B,C) show intermediate behavior, with reduced resistance relative to the pristine HGO, but not as pronounced as that of the AuNP/HGO sensor. In contrast, the AuNP/HGO sensor (Figure 5D) displays the smallest semicircle radius across all humidity levels, revealing the lowest overall resistance and suggesting the most efficient charge conduction among the devices analyzed.^52,53^

**Figure 5:**
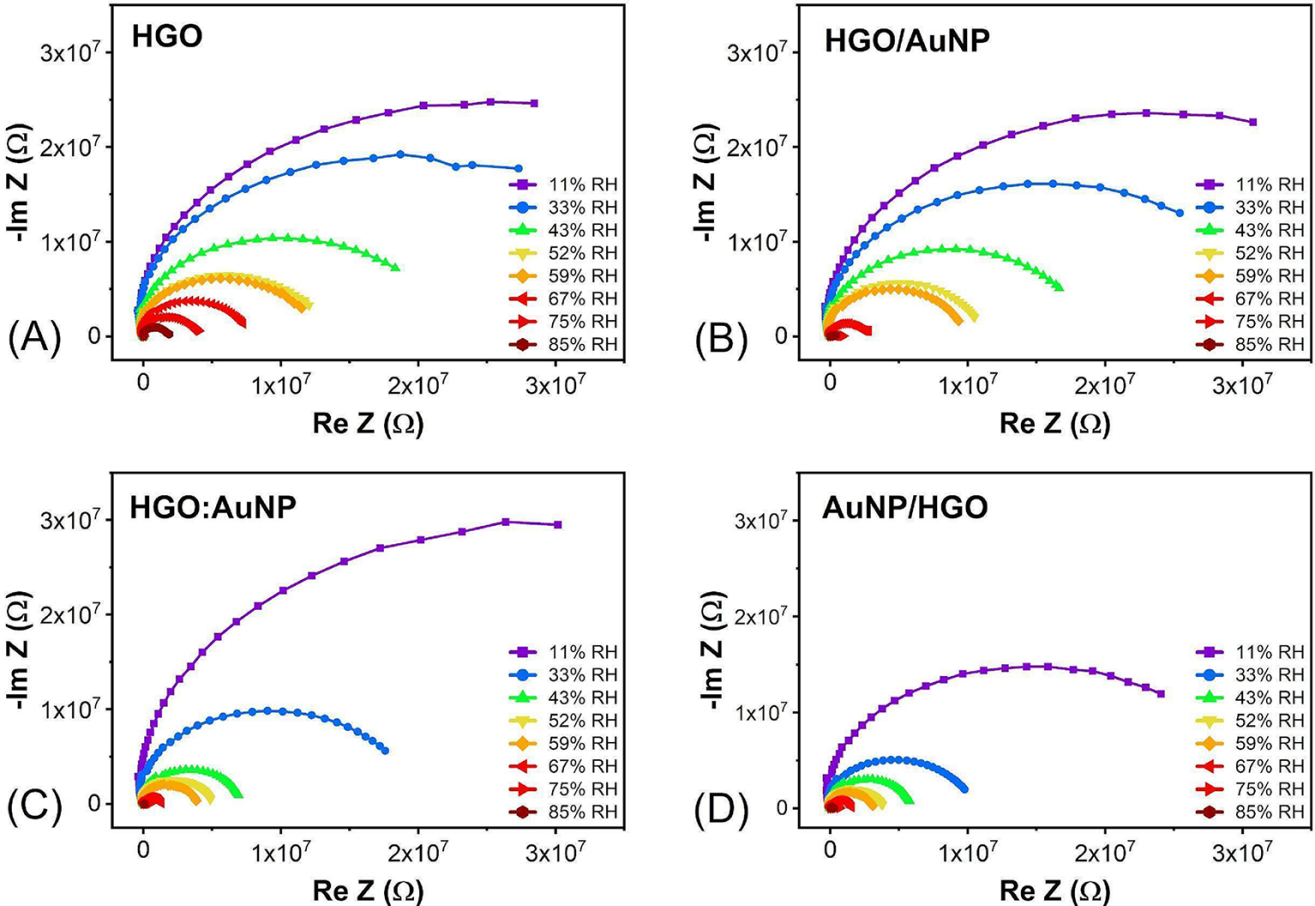
Complex impedance spectroscopy using a Nyquist plot for each sensor. (a) HGO, (b) HGO/AuNP, (c) HGO:AuNP, and (d) AuNP/HGO.

The semicircular behavior is associated with the conduction of protons (H^+^) jumping between water molecules and forming hydronium ions (H_3_O^+^) via the Grotthuss mechanism.^54–58^ As shown in Figure 6A, at low humidity levels, few water molecules adhere to the HGO surface, resulting in chemically adsorbed water monolayers that are insufficient to enable conduction through proton hopping due to the high activation energy required.^13^ As illustrated in Figure 6B, as the RH increases, additional water layers are formed through electrostatic attraction to the OFGs, facilitating proton jumps due to the reduced activation energy. Furthermore, the electric field generated by an external voltage induces polarization of water molecules, enhancing hydrogen bonding between them and consequently increasing H_3_O^+^ production. Concurrently, with the increase in RH, water begins to intercalate between the HGO layers, increasing the interlayer distance and intensifying the formation of the Grotthuss chain, as depicted in Figure 6C. This schematic representation illustrates the proposed humidity sensing mechanism, based on the impedance results and supported by the literature. The formation of hydrogen-bonded water networks enables proton conduction between hydroxyl groups via a Grotthuss-type mechanism, which explains the observed decrease in impedance under humid conditions.

**Figure 6:**
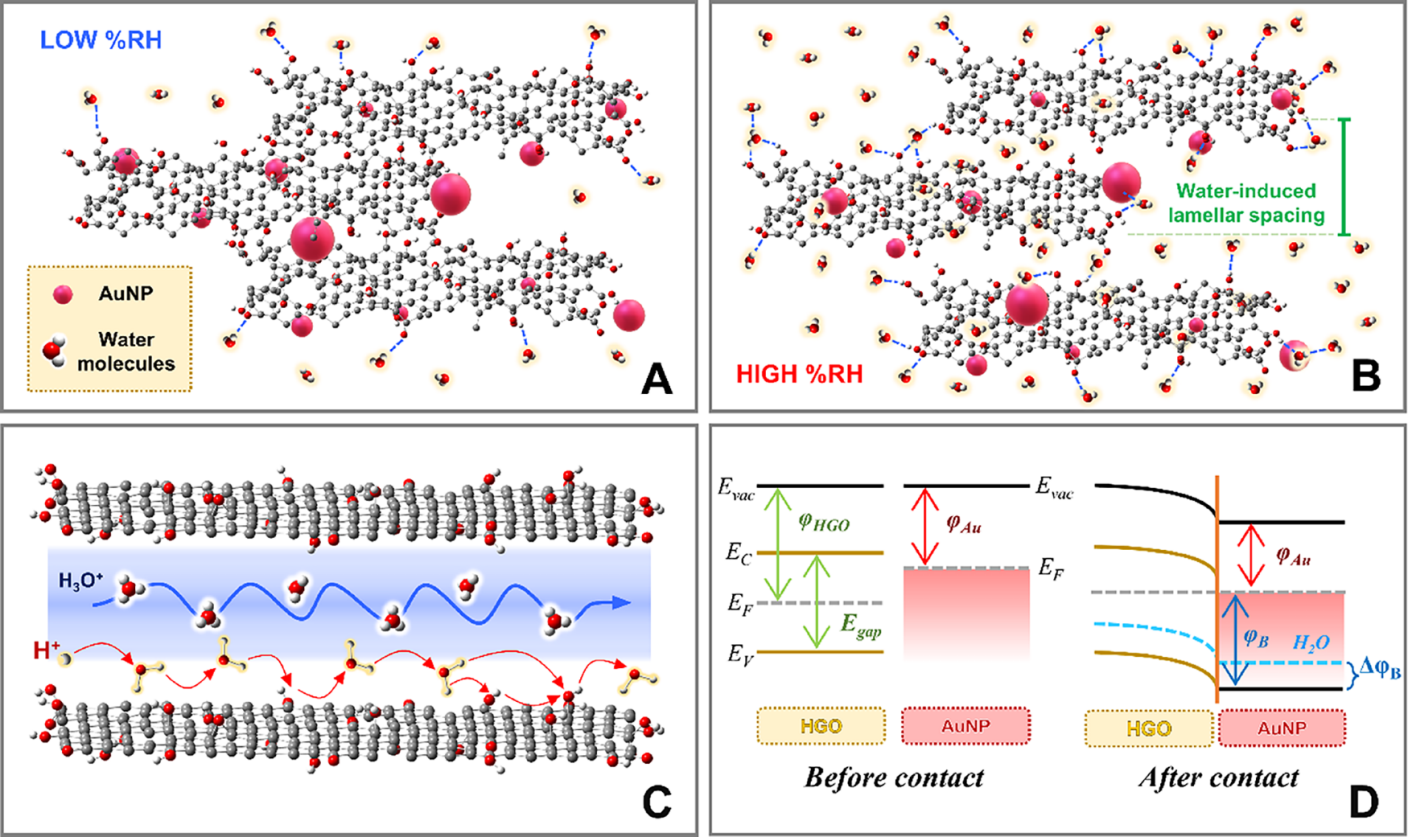
Schematic representation for the interaction of water molecules with HGO/AuNP structures under (A) low and (B) high RH. (C) Schematic representation of the Grotthuss mechanism. (D) Energy diagram for HGO and AuNP before and after its physical contact and after water adsorption.

In addition to the decrease in the radius of the semicircle, analysis of the impedance at 85% RH, as highlighted in Figure 7A–D, revealed straight lines connecting the end of the semicircle for devices containing AuNPs. The equivalent circuits shown in Figure 7E,F were selected based on the distinct impedance behaviors observed under different humidity levels. At low RH, the response can be modeled using a simple parallel resistor-capacitor configuration, associated with charge transfer and interfacial capacitance. However, at high RH, especially in sensors containing AuNPs, ionic diffusion contributes significantly to the overall impedance. To account for this effect, a Warburg element was incorporated into the circuit model in Figure 7F, reflecting the diffusion-controlled transport of ions through interlayer regions and surface pathways. Figure 7E illustrates the equivalent circuit at all RH values for the HGO sensor and at low humidity for the sensors with AuNPs. Figure 7F shows the corresponding circuit for the sensors with AuNPs at high RH, facilitating a deeper investigation of this anomalous behavior. Under high RH conditions, ion diffusion also occurs along with the Grotthuss mechanism, with H_3_O^+^/H^+^ moving freely on the surface and between HGO layers, substantially increasing conduction. This diffusion is directly linked to the Warburg impedance, which is strongly related to the ion transport behavior on the GO surface.^59,60^ All sensors exhibit proton-jumping conduction; however, only sensors with metallic NPs display Warburg impedance due to ionic diffusion, which becomes even more evident under high RH conditions. This additional conduction is particularly pronounced in the HGO:AuNP sensor, as shown in Figure 7C.

**Figure 7:**
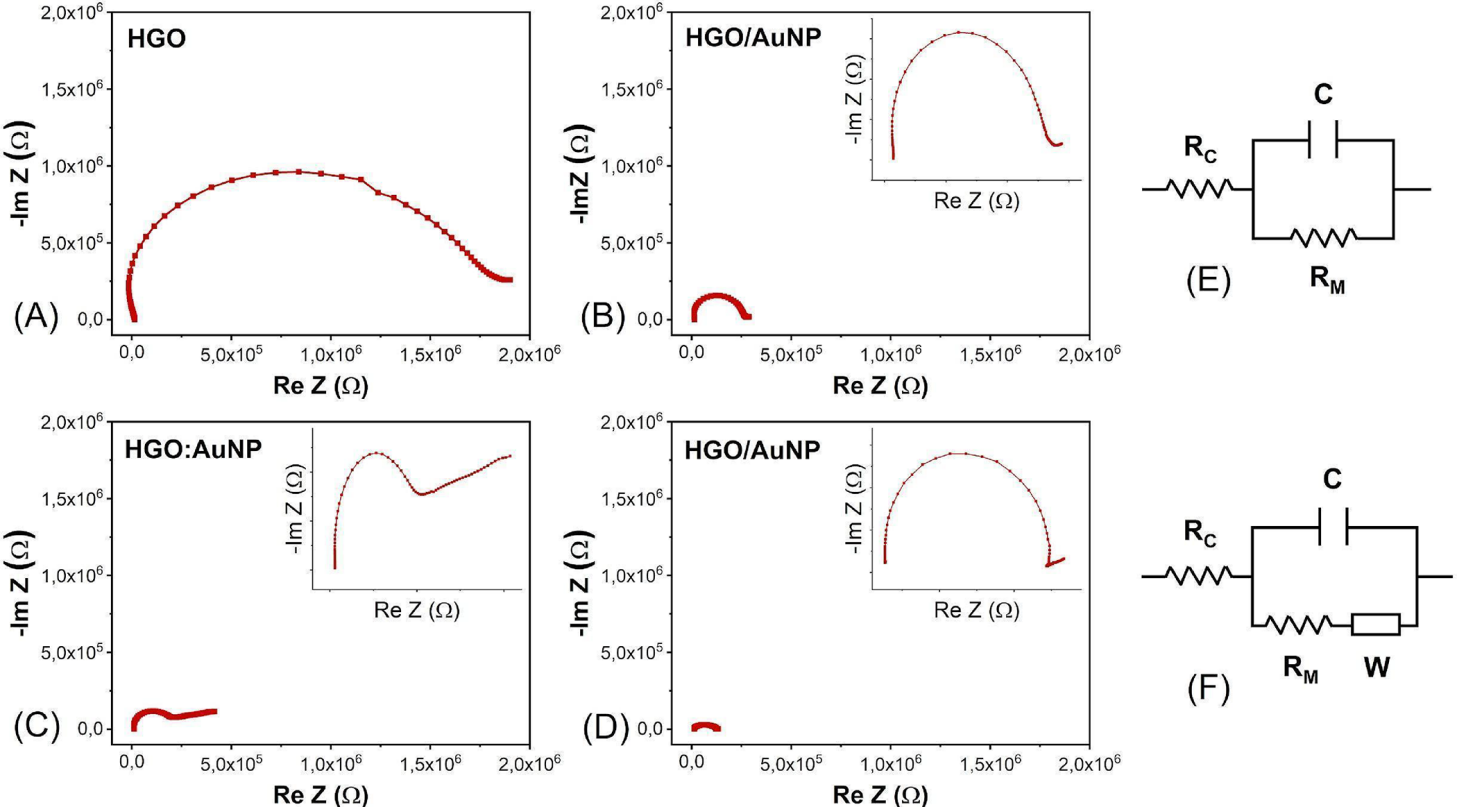
Complex impedance spectroscopy using Nyquist plots at 85% RH for (A) HGO, (B) HGO/AuNP, (C) HGO:AuNP, and (D) AuNP/HGO. (E, F) Sensor equivalent circuits under low and high RH conditions, respectively; RC: contact resistance; RM: material resistance; C: capacitance; W: Warburg impedance.

A phenomenon that directly influences these properties is the Schottky junction, represented in Figure 6D. This Schottky barrier originates from the difference in work functions between AuNPs (∼5.1 eV) and graphene oxide (∼4.7 eV), which leads to Fermi level alignment and the formation of a built-in potential at the interface.^61^ As a result, the barrier opposes charge carrier flow from GO to AuNPs, reducing interfacial conductivity, especially under low humidity conditions, where fewer charge carriers are available. Nevertheless, under high RH conditions, the excess water molecules, polarized by the applied electric field, tend to attract inner electrons to the surface of the material, effectively reducing the work function. Consequently, this reduction in work function lowers the potential barrier and increases sensor conductivity.^27,28^ Therefore, understanding the role of humidity in modulating the electrical properties in Schottky junctions is essential for developing humidity sensing devices.

Furthermore, with regard to the influence of resistance in the electrical analyses, the capacitive effects of the sensors are also considered. The capacitance of the sensors is related to a parallel plate capacitance scheme, as described by eq 1:

$$C=\varepsilon_{0}\varepsilon_{r}\frac{A}{d}$$ 1

where ε_0_ is the permittivity of free space (8.854 × 10^–12^ F m^–1^), ε_r_ is the relative permittivity of the dielectric material, and *A* and *d* are the surface area and the distance between the parallel plates, respectively; in our case, the HGO layers act as these plates. When water is inserted between the layers, there is an increase in the distance between them (increase in *d*), leading to a decrease in capacitance. Nonetheless, as RH increases, water itself becomes a dielectric medium, raising ε_r_ and thus the capacitance. In our study, these two effects counterbalance each other, resulting in negligible changes in capacitance values. Regarding capacitance, the frequency corresponding to the peak of the semicircle is considered for the calculation. Although the capacitance values for the AuNP/HGO sample (4.1–11.4 pF) differ slightly from those of the HGO device (8.8–11.5 pF), this variation is relatively minor compared to the substantial change in resistance observed under different humidity levels. Thus, capacitance appears to play a limited role in the sensing mechanism, with the dominant contribution arising from resistance modulation. In Figure S1, the behavior of the contact resistance, material resistance, and capacitance as a function of humidity is detailed for each sensor. As shown in Figure S1, while contact resistance remains relatively unchanged, the film resistance exhibits significant variation with humidity, confirming its primary role in the sensing response. The relatively stable capacitance supports the interpretation that water-induced changes in dielectric properties compensate for increased interlayer spacing, resulting in a negligible impact on the overall impedance. Also, Figure S2 shows the behavior of impedance as a function of frequency. It is observed that significant impedance changes with RH are noticeable only at low-frequency values. For AuNP-based sensors, shown in Figure S2B–D, the impedance changes as a function of RH are strongly shifted to higher frequencies compared to the HGO sensor. At higher frequencies, the impedance tends to approach contact resistance, while at low-frequency values, the impedance is dominated by the film resistance. When a high-frequency alternating voltage is applied, water molecules are less affected by changes in the electric field, reducing RH detection efficiency.^62^ Therefore, an impedance humidity sensor usually must be operated at a suitable working frequency. Figure S3A–D depicts the logarithm of impedance as a function of RH at frequencies of 100, 500, 1000, 2000, and 5000 Hz. These frequencies were selected to examine the behavior of the devices across the entire RH range. Upon analyzing the data, we observe that humidity detection improves as the frequency decreases, which also aligns with other works reported in the literature.^63–65^

Therefore, the sensors were analyzed at the optimal working frequency of 100 Hz, and their sensitivities were determined by eq 2

$$S=\frac{\log\;Z_{\max}-\log\;Z_{\min}}{{\mathrm{RH}}_{\max}-{\mathrm{RH}}_{\min}}$$ 2

where *Z*_max_ and *Z*_min_ represent the maximum and minimum impedance, and RH_max_ and RH_min_ denote the maximum and minimum RH, respectively. Figure 8A shows the impedance dependence on RH values for all sensors including their respective equations and linearity values. The performance of the sensors in this work is detailed in Table 1 and can be compared with similar devices reported in the literature. The results summarized in Table 1 highlight the relevance of the sensitivity values obtained in this study, enabling a direct comparison with those of previously reported GO-based humidity sensors. This comparative analysis underscores the effectiveness of our approach and demonstrates that while different methodologies may report sensitivity using various metrics, the values obtained here are within a competitive range. These results reinforce the practical significance of our sensor design and its favorable performance within the broader context of humidity sensing technologies. The linear adjustment of the logarithm of the resistance of the devices shows good linearity with *R* > 0.91 in the range from 11 to 85%RH. To provide a clearer visualization, Figure 8B presents a bar graph depicting the sensitivities of each sensor. As expected, the HGO sensor exhibited the lowest conductivity among all of the sensors. Conversely, the Au/HGO sensor demonstrated the highest detection capability, as evidenced by the significant decrease in impedance values with increased RH. The HGO, HGO/AuNP, HGO:AuNP, and AuNP/HGO sensors presented high sensitivity values of 0.016 ± 0.001, 0.023 ± 0.001, 0.024 ± 0.001, and 0.032 ± 0.001 log Z/%RH, respectively. Despite the electric field generated by the applied potential difference having the same effect in all deposition methods, the AuNP/HGO sensor stood out due to the direct contact of AuNPs with the thin aluminum film. Several studies show that the interaction between these materials directly influences charge movement within the structure,^66–68^ thereby justifying the higher sensitivity of this sensor. In the other two sensors, despite employing different deposition methods, interaction occurs solely between HGO and AuNPs, resulting in similar detection capabilities between them. As shown by Moskovits et al.,^69^ nanostructures of coinage metals such as Au can produce an intensified photoelectron emission phenomenon when excited by an external energy source, such as an applied alternating voltage, compared to the bulk form of the same metal. This phenomenon likely increases system current, enhancing charge transfer from nanoparticles and increasing sensitivity in Au-containing sensors.

**Figure 8:**
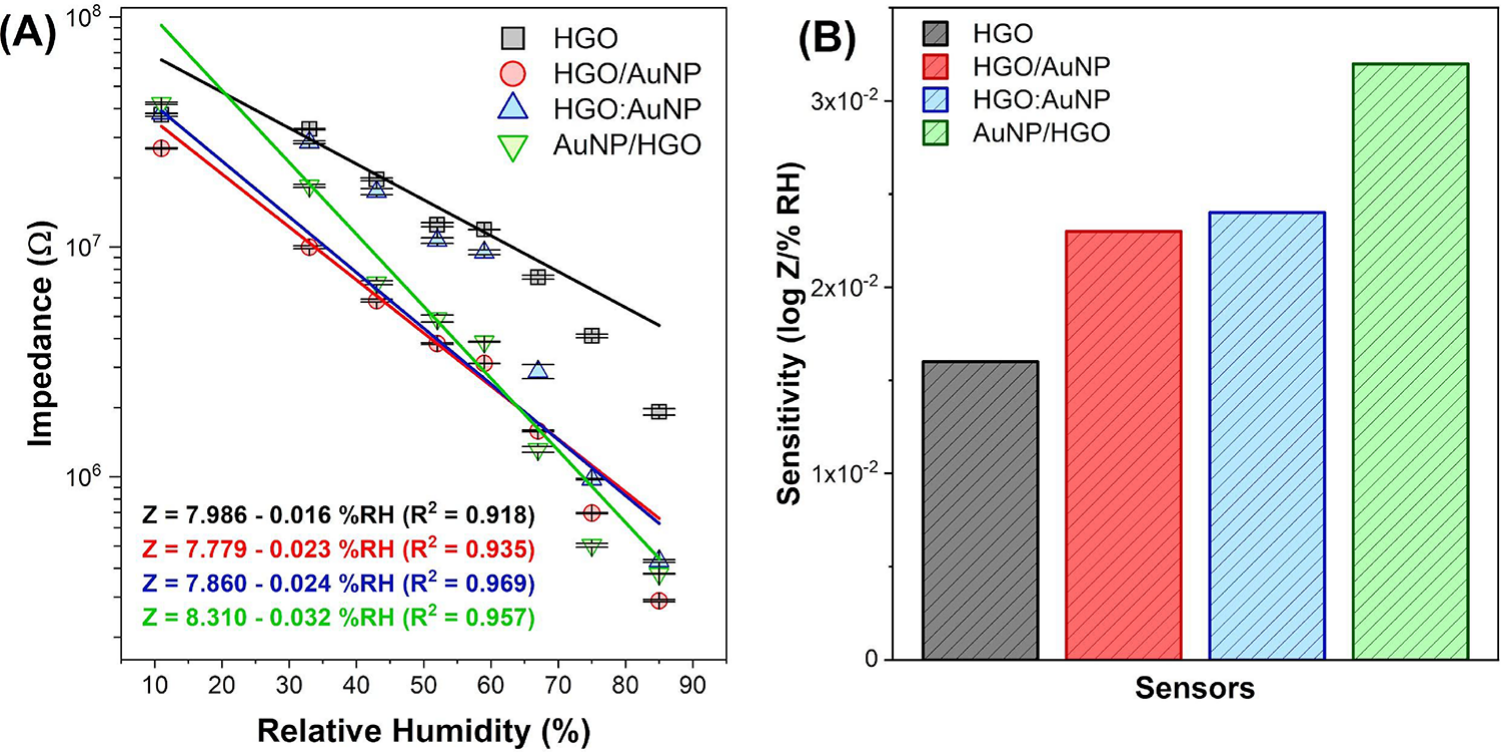
(A) Logarithm of impedance as a function of relative humidity for each humidity sensor manufactured at a working frequency of 100 Hz and its linear adjustments. (B) Sensitivities of each sensor determined at a frequency of 100 Hz.

**Table 1:** Comparison with Other Humidity Sensors Found in the Literature

materials	type	sensitivity	detection range	reference
HGO	impedance	0.016 log Z/%RH	11–85% RH	this work
HGO/AuNP (Au: 1.72 wt %)	impedance	0.023 log Z/%RH	11–85% RH	this work
HGO:AuNP (Au: 1.72 wt %)	impedance	0.024 log Z/%RH	11–85% RH	this work
AuNP/HGO (Au: 1.72 wt %)	impedance	0.032 log Z/%RH	11–85% RH	this work
GO:Ag (Ag: 2 wt %)	capacitance	2.6 × 10^4^ pF/%RH	11–97% RH	^28^
Au/GO (Au: 9 wt %)/MPTMOS	impedance	0.028 log Z/%RH	20–90% RH	^29^
SnS_2_/GO	impedance	6.5 × 10^4^ Ω/%RH	11–97% RH	^56^
ZnO/GO	capacitance	1.8 × 10^4^ pF/%RH	0–97% RH	^62^
Au nanospheres	impedance	0.038 log Z/%RH	10–95% RH	^68^
diamine-functionalized GO	impedance	0.054 log Z/%RH	20–90% RH	^70^

Figure 9A,B presents the response and recovery times of the HGO and Au/HGO sensors, respectively, measured by briefly exposing the sensors to breath while also open to the atmosphere. In graphene oxide-based humidity sensors, the response time is defined as the duration the sensor takes to register a measurable change in impedance after exposure to an increase in relative humidity. The recovery time refers to the period required for the impedance to return to its initial state after the humidity source is removed. These parameters reflect the sensor’s dynamic behavior and are critical for assessing its real-time applicability in humidity monitoring.

**Figure 9:**
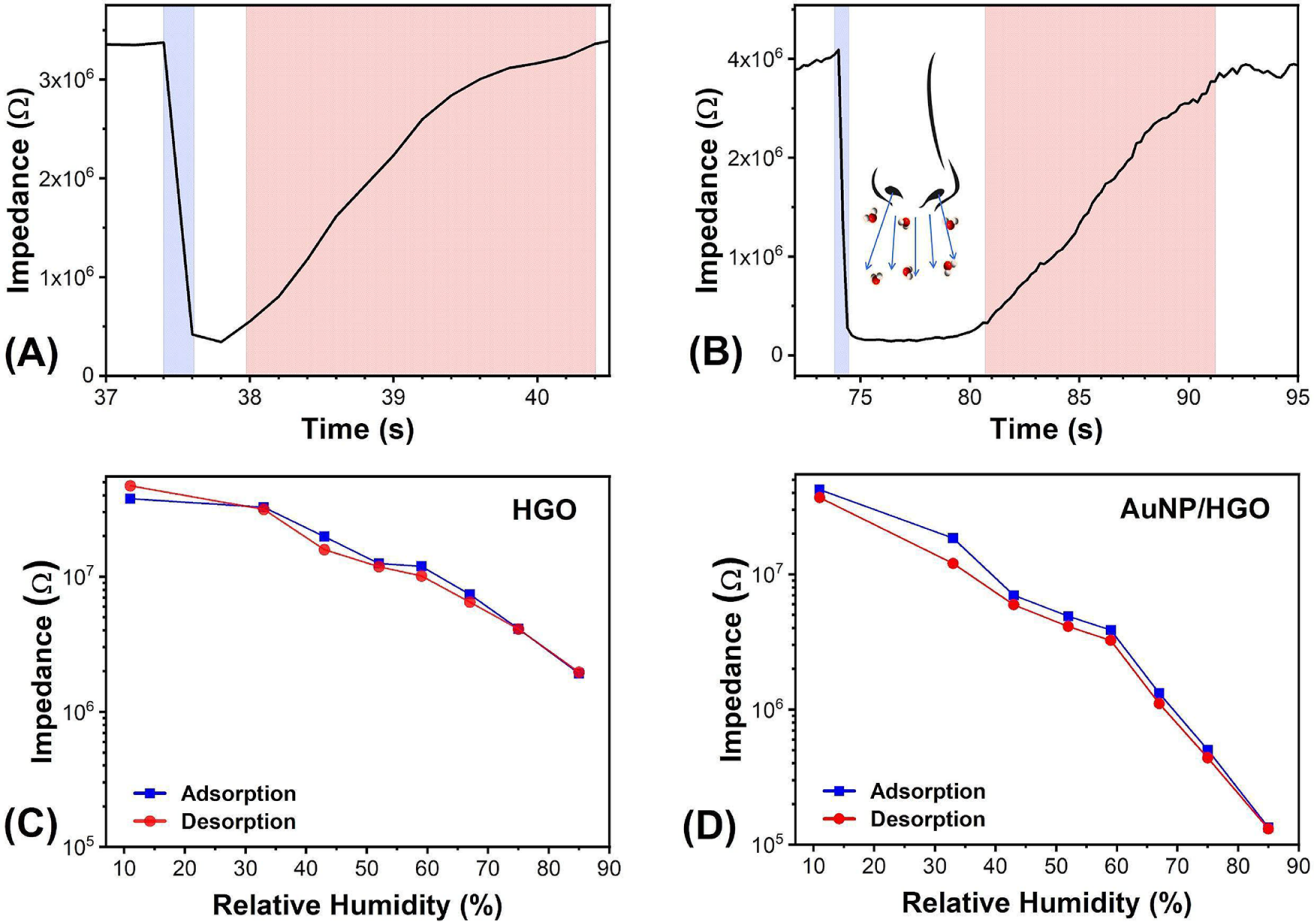
Response and recovery times of (A) HGO and (B) Au/HGO sensors. Hysteresis profile during the adsorption–desorption process for (C) HGO and (D) Au/HGO sensors.

The measurement was conducted in this manner to monitor human respiration. The HGO sensor exhibited a response time of approximately 0.2 s and a recovery time of 2.0 s. In the Au/HGO sensor, response and recovery times of 0.8 and 12.6 s, respectively, were observed. Figure 9C,D shows the hysteresis behavior in the adsorption–desorption process of the HGO and Au/HGO sensors. In this analysis, there are no significant changes in the hysteresis profile. Nonetheless, the small variation at low RH is mainly attributed to the excellent water sorption capability of GO-based materials, which consequently makes water release more difficult.

Along with sensitivity, hysteresis, time response, and recovery, stability is a crucial parameter for evaluating the humidity detection properties and durability of a material, especially for potential commercial applications. To assess stability, the impedance values of HGO and Au/HGO sensors were tested under fixed humidity levels over a 40-day period. The results shown in Figure S4, which compare the impedance values of the HGO and AuNP/HGO sensors at three relative humidity levels (11, 52, and 85%) measured on Day 1 and after 40 days, demonstrate excellent performance stability for both configurations. Over time, both sensors maintained consistent impedance responses, confirming their long-term reliability. Notably, the AuNP/HGO sensor exhibited consistently lower impedance values compared to those of the HGO sensor, indicating enhanced conductivity and improved interfacial charge transfer. These findings reinforce the superior sensing performance of the AuNP/HGO design and its suitability for prolonged operation in humidity detection applications.

In addition to the impedance response as a function of relative humidity, complementary analyses were carried out to evaluate the dynamic performance and stability of the devices. In the real-time impedance measurements, the AuNP/HGO sensor (Figure S5B) exhibited a significantly more pronounced decrease in impedance in response to increasing humidity compared to the sensor based solely on the HGO sensor (Figure S5A), confirming its enhanced sensitivity. Moreover, the incorporation of AuNPs resulted in a considerable reduction in signal noise during data acquisition, producing a more stable and well-defined electrical response—an important characteristic for practical sensing applications.

For the repeatability analysis (Figure S5C,D), successive humidity cycles were performed between low (11% RH) and high humidity conditions (85% RH). Both sensors demonstrated consistent and reproducible responses throughout the cycles, confirming good operational stability. However, the sensor containing AuNPs showed sharper transitions and lower signal dispersion between cycles, whereas the HGO-only sensor, although functional, exhibited higher noise levels that may hinder precise response interpretation. These findings further reinforce that the incorporation of gold nanoparticles not only enhances sensitivity but also improves the sensor’s stability and signal quality during operation.

## 3 Conclusions

This study primarily focused on the impedance response of hydroxyl-rich hydrogenated graphene oxide (HGO) modified with gold nanoparticles (AuNPs) for humidity sensing applications. The results demonstrated that AuNP modification significantly enhances the electrical properties of HGO, including increased electrical conductivity and reduced charge transfer resistance. These improvements arise from the excellent conductivity of AuNPs, which facilitates electron mobility and introduces additional conductive pathways within the HGO matrix. Additionally, the influence of sensor architecture was explored, and it was found that the configuration in which AuNPs are first deposited onto the aluminum interdigitated electrodes leads to superior sensing performance. This enhancement is attributed to improved interfacial contact and more efficient charge transport between the electrode and the sensing layer. Hysteresis tests revealed no significant changes for both HGO and AuNP/HGO sensors, with the material exhibiting excellent adsorption–desorption behavior. Both the HGO and AuNP/HGO sensors demonstrated rapid adsorption response times and satisfactory desorption times, in agreement with typical values reported for similar graphene oxide-based platforms. These characteristics make them suitable candidates for practical humidity monitoring applications. Impedance analyses facilitated the examination of the resistive and capacitive properties of each device, revealing resistive behavior with variations in relative humidity alongside consistent capacitance values. The humidity sensing mechanism was thoroughly investigated in light of the experimental findings, revealing that both proton conduction and ionic diffusion play central roles in sensor operation. This conclusion is supported by the Nyquist plots, which show semicircular arcs related to proton hopping and linear low-frequency behavior indicative of Warburg impedance. The effect of ionic diffusion was more evident at higher RH and in AuNP-containing devices, as illustrated in Figure 7. This study provides significant evidence that, contrary to common reports in the literature, the enhanced sensitivity of the sensor arises not only from increased water adsorption but also from improved charge detection due to the modulation of the Schottky barrier induced by adsorbed water molecules. This effect is supported by impedance measurements, which reveal a clear decrease in charge transfer resistance with increasing relative humidity—indicative of barrier lowering at the interface. The influence of water adsorption on the Schottky barrier was further corroborated by the use of different sensor fabrication methods, particularly through the inversion of material deposition layers.

## 4 Experimental Section

### 4.1 Materials

Sulfuric acid (95–98%, H_2_SO_4_, Qhemis), graphite powder (98%, Synth), potassium permanganate (99%, KMnO_4_, Synth), hydrogen peroxide (30%, H_2_O_2_, Sigma-Aldrich), hydrochloric acid (37%, HCl, Qhemis), isopropyl alcohol (99%, C_3_H_8_O, EasyPath), tetrachloroauric acid hydrate (99%, HAuCl_4_·*x*H_2_O, Sigma-Aldrich), sodium borohydride (≥99%, NaBH_4_, Sigma-Aldrich), hydroxylamine hydrochloride (99%, NH_2_OH·HCl, Sigma-Aldrich), aluminum pellets (99.99%, 1/8″ diameter × 1/8″ long, Al, Kurt J. Lesker), lithium chloride (≥99%, LiCl, Merck), magnesium chloride (99%, MgCl_2_, Synth), potassium carbonate (98%, K_2_CO_3_, Vetec), magnesium nitrate (99%, Mg­(NO_3_)_2_·6H_2_O, Sigma-Aldrich), sodium bromide (99%, NaBr, Synth), copper chloride (99.8%, CuCl_2_·2H_2_O, Sigma-Aldrich), sodium chloride (99%, NaCl, Vetec), and potassium chloride (99%, KCl, Vetec) were used. All glassware was cleaned using fresh aqua regia and thoroughly washed with deionized water and isopropyl alcohol. All reactants were used without further purification, and all solutions were freshly prepared with Milli-Q water (18.2 MΩ cm resistivity at 25 °C).

### 4.2 Instrumentation

UV–vis spectra were acquired with an Ocean Optics USB 2000+ spectrometer by using quartz cuvettes with a 0.5 cm optical path length. Hydrodynamic diameter analysis was performed on a Malvern Nano ZS90 Dynamic Light Scattering (DLS) instrument, operating with a He–Ne laser at a wavelength of 632.8 nm, a 90° detection angle, and a 1.0 cm optical path length in glass cuvettes. Raman spectra were collected on a Bruker SENTERRA Raman spectrometer, equipped with a charge-coupled device detector, an Olympus BX51 microscope, and a 532 nm excitation laser line from a frequency-doubled Nd laser. All spectra were acquired with a 50× long working-distance objective, using a nominal laser power of 0.2 mW. Transmittance FT-IR spectra were obtained with a Bruker Vertex 70 instrument and an attenuated total reflectance accessory, operating in the range of 4000 to 400 cm^–1^, with a spectral resolution of 4 cm^–1^. SEM measurements were conducted using an FEI Quanta 250 microscope at 20 kV under high-vacuum conditions (pressure range: 10^–5^ to 10^–6^ mbar). Tapping-mode AFM measurements were made using a Park System NX10 instrument with a silicon tip at a frequency of 70 kHz. Impedance measurements were performed using an Ivium Technologies CompactStat potentiostat/galvanostat in a two-electrode configuration, with a frequency range from 10^2^ to 10^6^ Hz and an amplitude of 0.01 V. Interdigitated aluminum electrodes were deposited in a resistive thermal evaporation chamber under a pressure of 10^–6^ mbar in an MBRAUN MB200 Glovebox.

### 4.3 Synthesis of Hydroxyl-Rich Graphene Oxide (HGO)

HGO was synthesized via a modified Hummers’ method,^71^ based on protocols by Chen et al.^19^ and De Lima et al.,^72^ as follows: 92.0 mL of concentrated H_2_SO_4_ was added to 24.0 mL of deionized water and stirred at 200 rpm in an ice bath until the temperature reached approximately 10 °C. Then, 2.0 g of graphite powder and 6.0 g of KMnO_4_ (added in three increments every 30 min) were incorporated. The mixture was heated to over 100 °C for 10 min, then cooled to 40 °C, maintaining that temperature for 2 h. Subsequently, 600.0 mL of deionized water was added to the reaction mixture, stirred for 20 min, followed by 10.0 mL of concentrated H_2_O_2_ to complete the oxidation. The product was washed with concentrated HCl and deionized water until a neutral pH was reached. GO aqueous suspensions were prepared by dispersing dried HGO in deionized water by using a tip sonicator.

### 4.4 Synthesis of Gold Nanoparticles (AuNPs)

Colloidal AuNPs were synthesized by adapting the Creighton reduction method^73^ as follows: 15.0 mL of a freshly prepared 2.0 × 10^–3^ mol L^–1^ NaBH_4_ solution was mixed with 50.0 μL of a 1.0 × 10^–1^ mol L^–1^ NH_2_OH solution and placed in an ice bath under vigorous stirring. To this mixture was added 5.0 mL of a freshly prepared 1.5 × 10^–3^ mol L^–1^ HAuCl_4_ solution, producing an immediate intense red color, characteristic of small AuNPs. The colloidal suspension was maintained in the ice bath under continuous vigorous stirring for 2 h to stabilize the NPs.

### 4.5 Fabrication of Sensors and Humidity Measurements

Soda-lime glasses were used as substrates for all sensors, fabricated by thermal evaporation. Prior to the deposition of interdigitated aluminum (Al) films, glass substrates (12.5 × 25 mm^2^) were thoroughly cleaned in a piranha etch bath (H_2_SO_4_, 7:3) at 80 °C for 30 min to remove contaminants. Substrates were then extensively rinsed with deionized water to eliminate any residual piranha solution and stored in isopropyl alcohol. For Al deposition, substrates were transferred to a glovebox with a mask to demarcate the deposition area. Deposition commenced once the chamber pressure reached approximately 10^–6^ mbar, forming thin Al films on glass substrates with dimensions of 7.0 × 7.0 mm^2^. Four distinct sensors were produced, each following a specific procedure. For the first sensor, 40.0 μL of a 1.0 mg mL^–1^ HGO aqueous suspension was deposited. For the second sensor, a layer of 40.0 μL of HGO was deposited, followed by an additional layer of 10.0 μL of the AuNP suspension (denoted HGO/AuNP). For the third sensor, a previously prepared HGO and AuNP mixture at a 4:1 v/v ratio was used (denoted as HGO:AuNP). Finally, for the fourth sensor, 10.0 μL of AuNP was applied, followed by 40.0 μL of HGO (denoted as AuNP/HGO). Depositions were performed by using the drop-casting method onto the interdigitated electrode, followed by vacuum oven drying of the sensor at 60 °C for 2 h. At the end, all sensors containing AuNPs showed a final concentration of about 1.72 wt % of Au.

## Supplementary Material



## Notes

Additional electrical characterization of the sensor; capacitance and resistance measurements, impedance spectra as a function of frequency, as well as durability, stability, and repeatability tests (PDF)
