# Supplementary material for: High-Performance Impedance Humidity Sensor Based on Au Nanoparticle-Modified Hydroxyl-Rich Graphene Oxide
Source: ACS Omega. 2025 Sep 19;10(38):43706–18. doi: 10.1021/acsomega.5c03897 (PMC12489656; doi:10.1021/acsomega.5c03897)
Supplement: Supplementary file 1 [file ao5c03897_si_001.pdf]

High-performance impedance humidity sensor based on Au nanoparticles-modified hydroxyl-rich graphene oxide

Iuri K. Machado[1], Rafael de Oliveira[2], Marina C. Totti[1], Nayton C. Vicentini1, Wesley W. G. Nascimento[3], Benjamin Fragneaud[1], Indhira O. Maciel[1], Cristiano Legnani[1], Antonio Carlos Sant'Ana[2], Welber G. Quirino[1]*

[1]Nanoscience and Nanotechnology Group, Department of Physics, Institute of Exact Sciences, Federal University of Juiz de Fora, Juiz de Fora, Minas Gerais, 36036-900, Brazil

[2]Plasmonic Nanostructures Laboratory, Department of Chemistry, Institute of Exact Sciences, Federal University of Juiz de Fora, Juiz de Fora, Minas Gerais, 36036-900, Brazil

[3]Department of Pharmacy, Institute of Life Sciences, Federal University of Juiz de Fora, Governador Valadares Campus, Governador Valadares, Minas Gerais, 35010-180, Brazil

*E-mail: wgquirino@ufjf.br

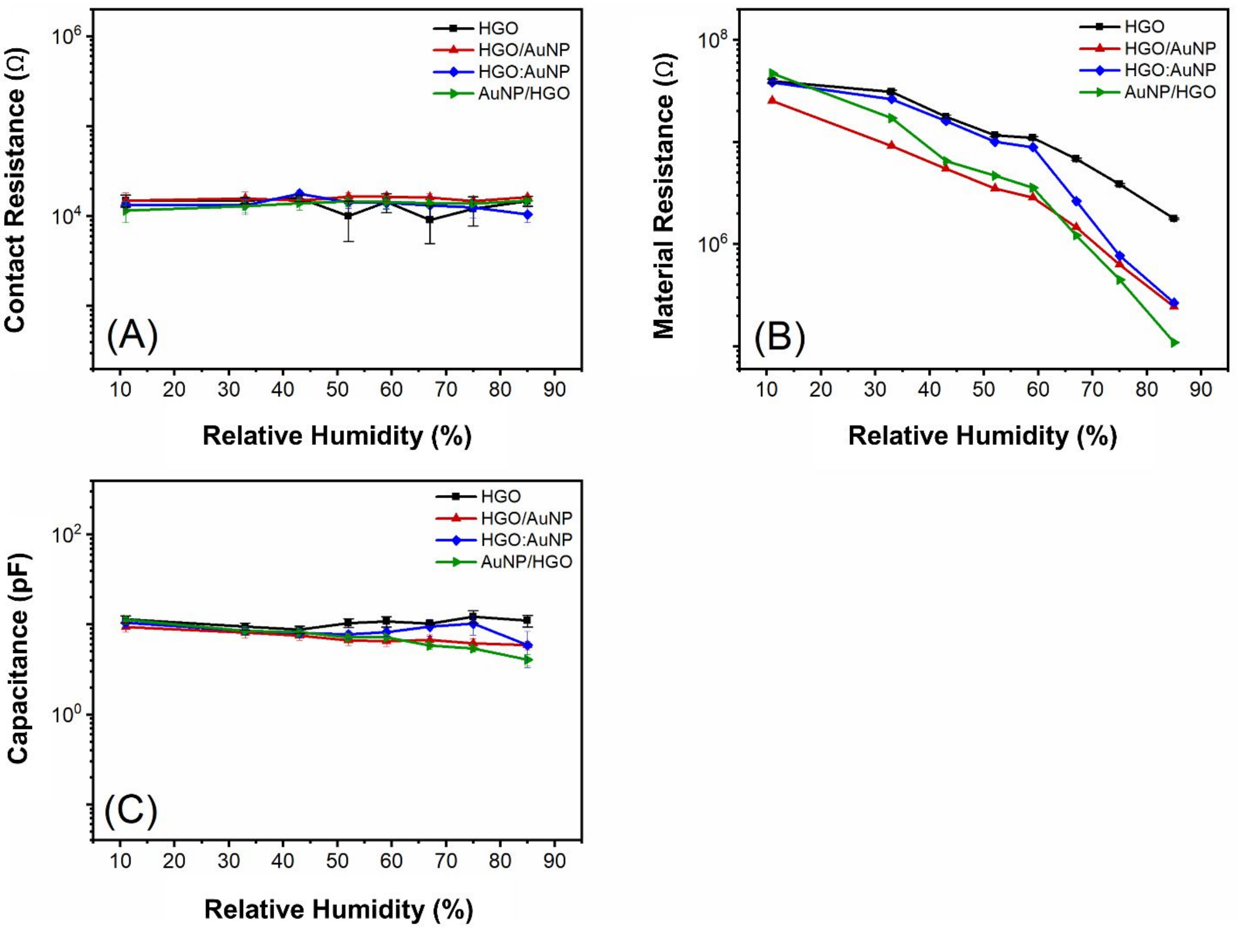

Figure S1. Behavior of contact resistance (A), material resistance (B), and capacitance (C) as a function of humidity for the four sensors.

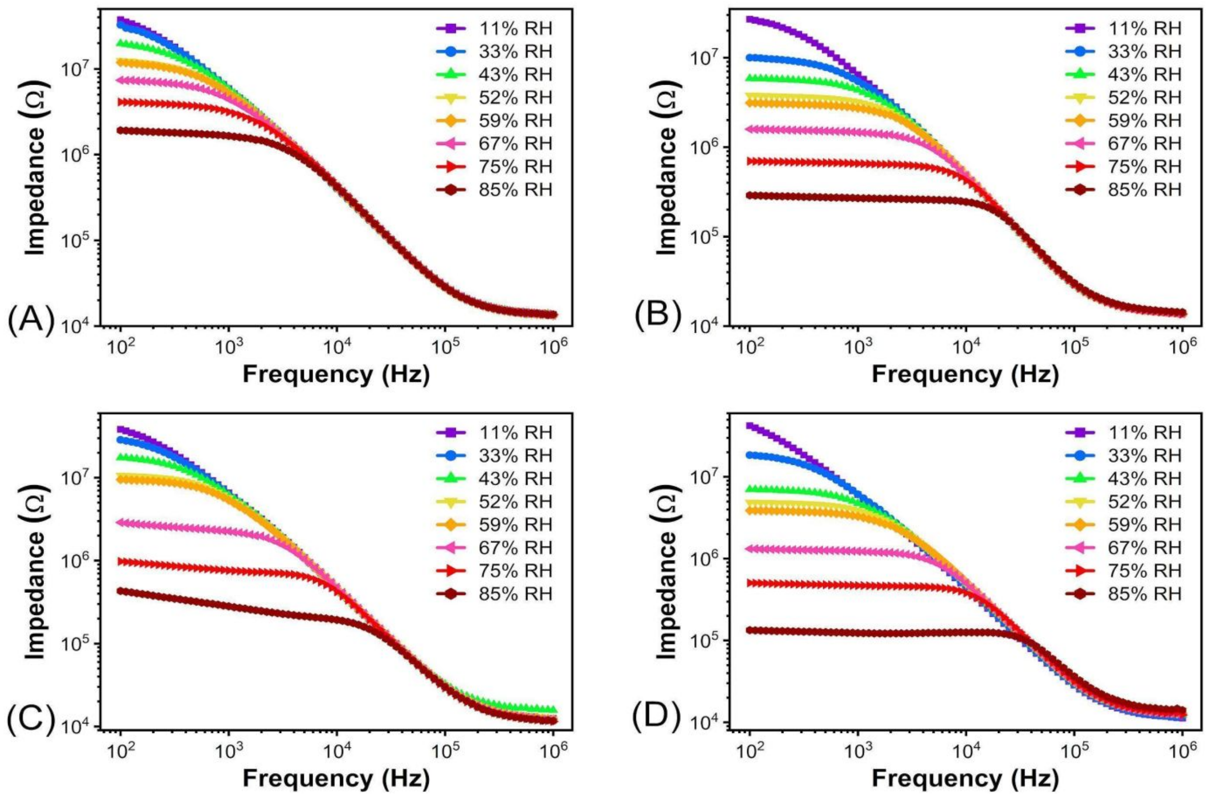

Figure S2. Bode plot for each sensor at different relative humidity: (A) HGO, (B) HGO/AuNP, (C) HGO:AuNP and (D) AuNP/HGO.

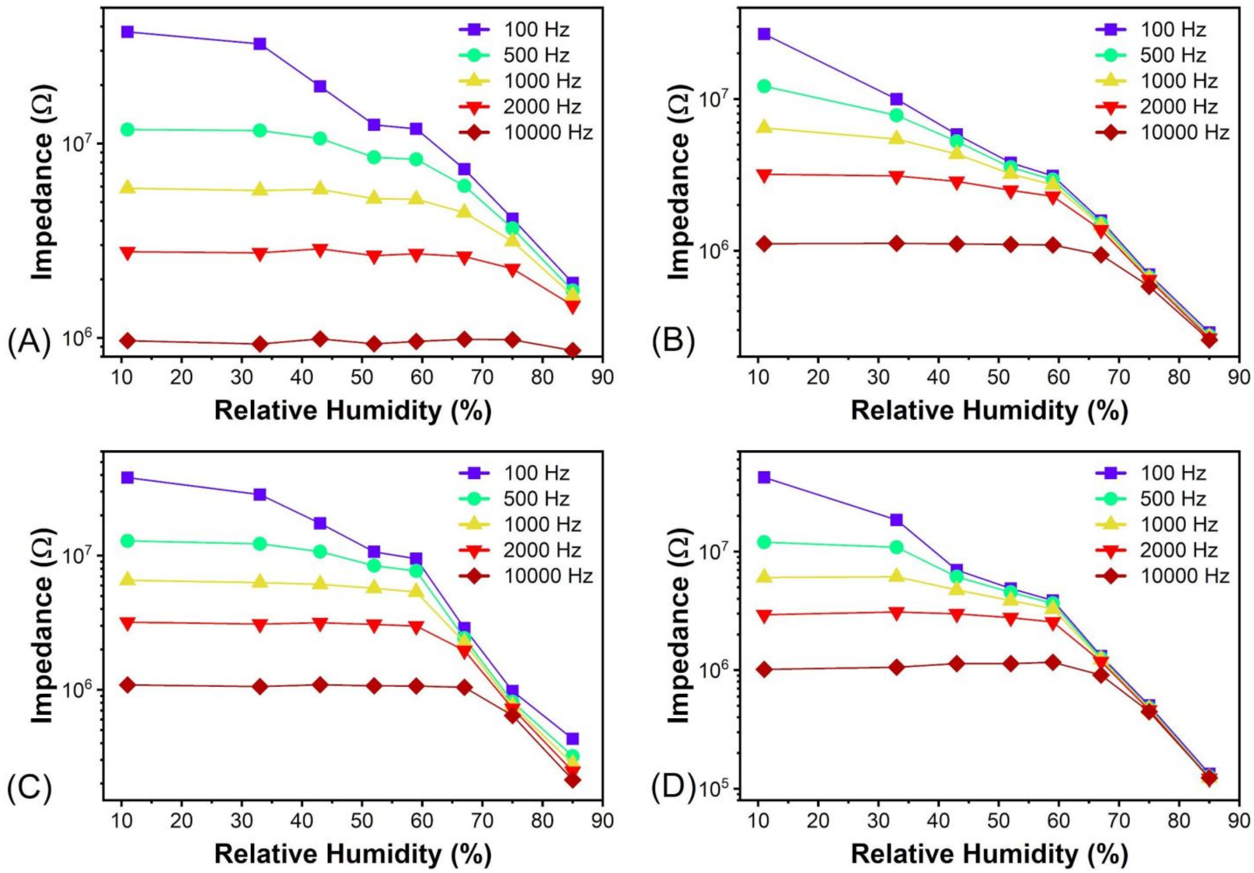

Figura S3. Impedance as a function of relative humidity for different frequency values in each sensor: (A) HGO, (B) HGO/AuNP, (C) HGO:AuNP and (D) AuNP/HGO.

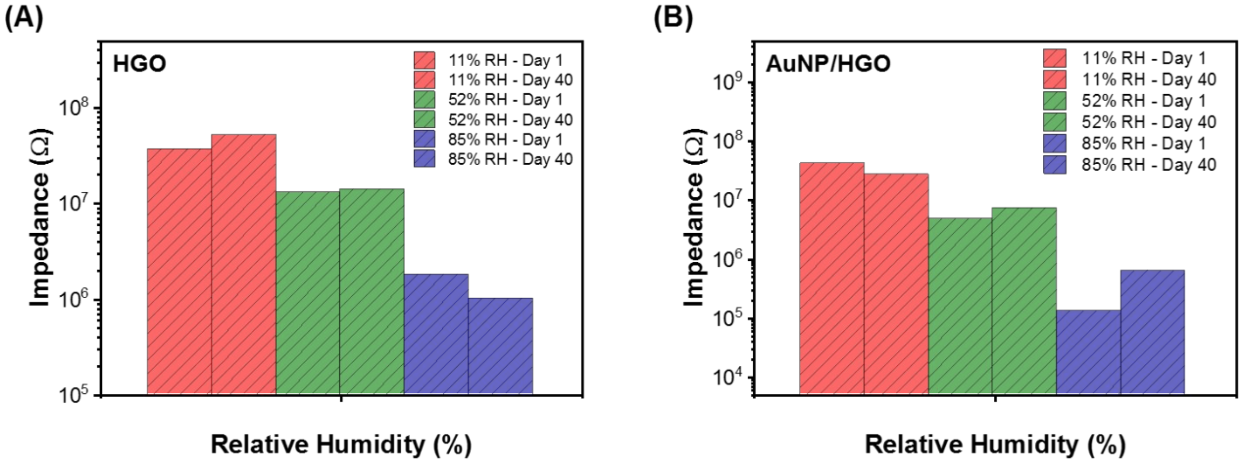

Figure S4. Stability of the (A) HGO and (B) AuNP/HGO sensor exposed to 11%, 52% and 85% RH

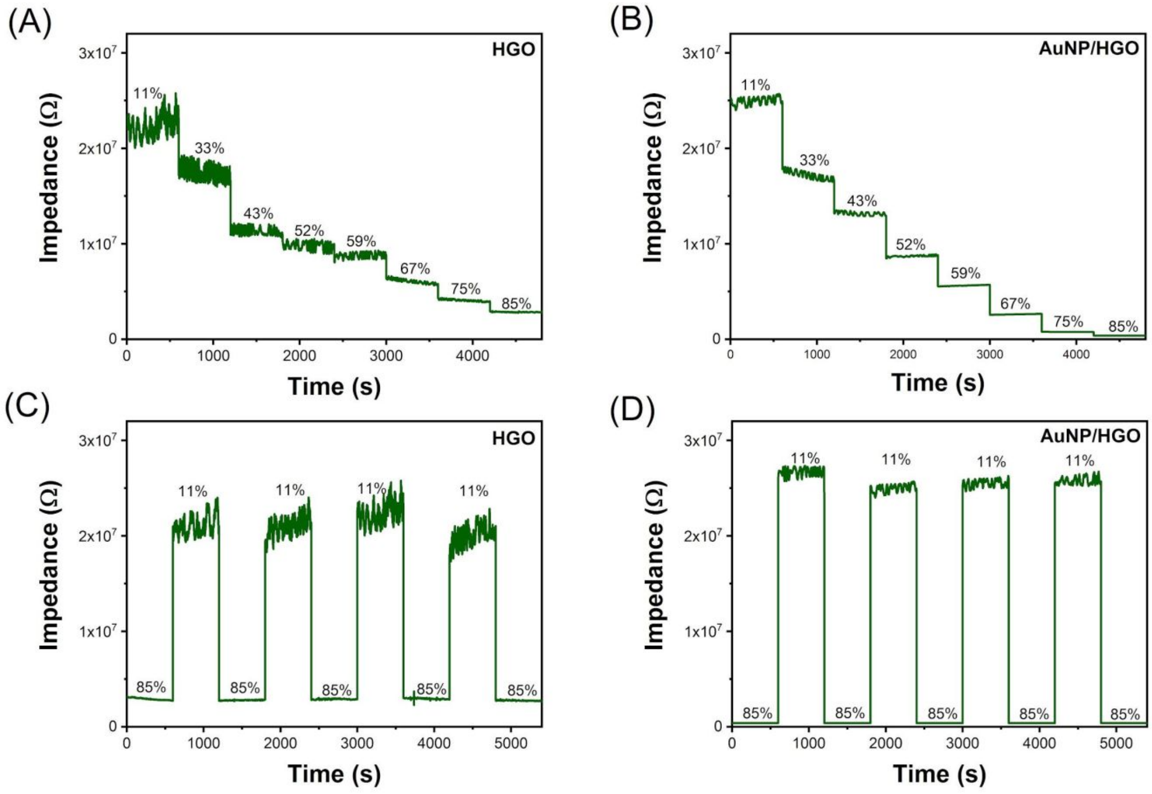

Figure S5. Real-time impedance response and repeatability analysis of the humidity sensors. (A) Impedance response of the HGO sensor as a function of time under stepwise increases in relative humidity. (B) Impedance response of the AuNP/HGO sensor under the same conditions, showing a more pronounced decrease in impedance and reduced signal noise. (C) Repeatability of the HGO sensor under cyclic exposure to low (11% RH) and high (85% RH) humidity, showing consistent

but noisier responses. (D) Repeatability of the AuNP/HGO sensor under the same humidity cycling, demonstrating more defined transitions and improved signal stability.
